# B‐EPIC: A Transformer‐Based Language Model for Decoding B Cell Immunodominance Patterns

**DOI:** 10.1002/advs.202508896

**Published:** 2025-10-07

**Authors:** Jun‐Ze Liang, Youtao Wang, Cong Sun, Tao Liu, Zengfeng Wu, Lipeng Chen, Lina Chen, Penglin Li, Zhengkang Li, Cangui Zhang, Bingyun Lu, Ye Chen, Bing Gu, Qian Zhong, Xin Wei Wang, Mu‐Sheng Zeng, Jinping Liu

**Affiliations:** ^1^ Department of Experimental Research State Key Laboratory of Oncology in South China Guangdong Provincial Clinical Research Center for Cancer Sun Yat‐sen University Cancer Center Guangzhou 510060 China; ^2^ Laboratory Medicine Guangdong Provincial People's Hospital (Guangdong Academy of Medical Sciences) Southern Medical University Guangzhou 510080 China; ^3^ Department of Gastroenterology Nanfang Hospital Southern Medical University Guangzhou Guangdong 510515 China; ^4^ Laboratory of Human Carcinogenesis Center for Cancer Research National Cancer Institute Bethesda MD 20892 USA; ^5^ Liver Cancer Program Center for Cancer Research National Cancer Institute Bethesda MD 20892 USA

**Keywords:** B cell epitope prediction, Immunodiagnostics design, pathogens prevention, transformer, vaccines development

## Abstract

Vaccine development for pathogens has faced significant challenges, contributing to a public health burden. B‐cell epitope (BCE) prediction is a crucial process in vaccine development, but is hindered by limited efficiency and accuracy. To address this, B‐Epic, the first pipeline applying Transformer to predict BCEs is independently developed. B‐Epic's robustness is validated through multiple testing datasets, including distinguishing clinically‐approved vaccine targets, identifying BCEs (the Immune Epitope Database testing dataset; *n* = 23,888) and immunoreactive peptides (*Trypanosoma cruzi* peptidome; *n* = 239,575) with high AUCs of 0.882 and 0.945, respectively, outperforming widely used tools. Based on its superior performance, B‐Epic is applied to the prevention of carcinogenic pathogens. In the application to *Helicobacter pylori*, peptides screened by B‐Epic can activate B cells in experiments, suggesting their potential as vaccine targets. In another application to Epstein‐Barr virus, B‐Epic identifies pan‐immunoreactive peptides in a clinical cohort (*n* = 899). These peptides exhibit higher reactogenicity in nasopharyngeal carcinoma patients than in healthy controls (*n* = 140), indicating their viability as immunodiagnostic targets. Overall, B‐Epic utilizes self‐attention, high‐dimensional feature projection, and convolutional neural networks to autonomously extract complicated BCE features, enabling accurate BCE prediction and thereby facilitating efforts to prevent infectious diseases and cancers.

## Graphical abstract

B‐Epic: Achieving high‐accuracy B cell epitope prediction through Transformer architecture. Comprehensive validation demonstrates B‐Epic's robust performance across diverse datasets. B‐Epic facilitates vaccine development for *H. pylori* and EBV, addressing critical public health challenges.

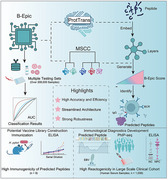

## 1 Introduction

Vaccines represent one of humanity's greatest medical triumphs, exemplified by the eradication of smallpox and the near‐elimination of several childhood diseases.^[^ ^1^, ^2^, ^3^ ^]^ Despite these historic successes, we face unprecedented challenges in effectively developing vaccines against complex pathogens that continue to impose substantial global health burdens.^[^ ^4^ ^]^ The persistent threat of Epstein‐Barr virus (EBV) is implicated in multiple malignancies, contributing to ≈240 000–358 000 new cancer cases annually; nevertheless, no licensed vaccines or immunodiagnostics exist after decades of research.^[^ ^5^, ^6^ ^]^ Similarly, *Helicobacter pylori* (*H. pylori*), which infects over 40% of the global population and significantly increases gastric cancer (GC) risk, remains without a preventive vaccine.^[^ ^7^, ^8^ ^]^

The rapid emergence of novel pathogens and their variants, coupled with sophisticated immune evasion mechanisms, poses unprecedented challenges to global public health security.^[^ ^9^, ^10^, ^11^, ^12^ ^]^ Traditional vaccine development approaches, though historically successful, are often too time‐consuming and resource‐intensive to be meet urgent demands.^[^ ^13^, ^14^ ^]^ Reverse vaccinology (RV), which integrates computer science with biology, is a primary approach in modern vaccine development. Utilizing omics data to identify B cell epitopes (BCEs), it significantly enhances the breadth of BCE identification and vaccine diversity. In the process, however, the expanding breadth of discovery exacerbates the challenge; hence, accurate and effective identification of BCEs from omics data is a major bottleneck in RV.^[^ ^15^, ^16^, ^17^, ^18^ ^]^

While experimental approaches such as Phage immunoprecipitation sequencing (PhIP‐seq) have revolutionized our ability to profile antibody‐antigen interactions at high throughput, they remain constrained by limited coverage and high resource consumption. These constraints are particularly problematic given the urgent need for rapid vaccine development in response to emerging pathogens.^[^ ^19^, ^20^, ^21^ ^]^

Traditional computational approaches for predicting BCEs have struggled to capture the complex molecular determinants of immunogenicity, resulting in high false‐positive rates that hamper efficient vaccine design.^[^ ^16^ ^]^ This challenge is especially acute for the development of precision diagnostics and therapeutics, where target specificity is paramount.^[^ ^22^, ^23^ ^]^

Recent advances in Transformers have transformed our ability to analyze biological sequences by understanding the “protein language”.^[^ ^24^ ^]^ These models excel at capturing both local and global sequence features through sophisticated high‐dimensional feature projection and self‐attention mechanisms, offering unprecedented capabilities in understanding amino acid (AA) patterns and their functional implications.^[^ ^25^ ^]^ Furthermore, Transformers directly utilize protein primary structure (PPS) as input, enabling a more streamlined architecture with enhanced efficiency and flexibility.^[^ ^26^, ^27^ ^]^

Here, we present B‐Epic, a novel Transformer‐based framework that achieves breakthrough performance in BCE prediction. Through comprehensive validation on diverse datasets containing over 250 000 peptides, B‐Epic demonstrated superior robustness in identifying BCEs across multiple pathogens. Our results establish a powerful new paradigm for accelerating BCE discovery, which facilitates vaccine development.

## 2 Results

### 2.1 Development of B‐Epic: A Transformer‐Based Approach for BCE Prediction

**Figure 1:**
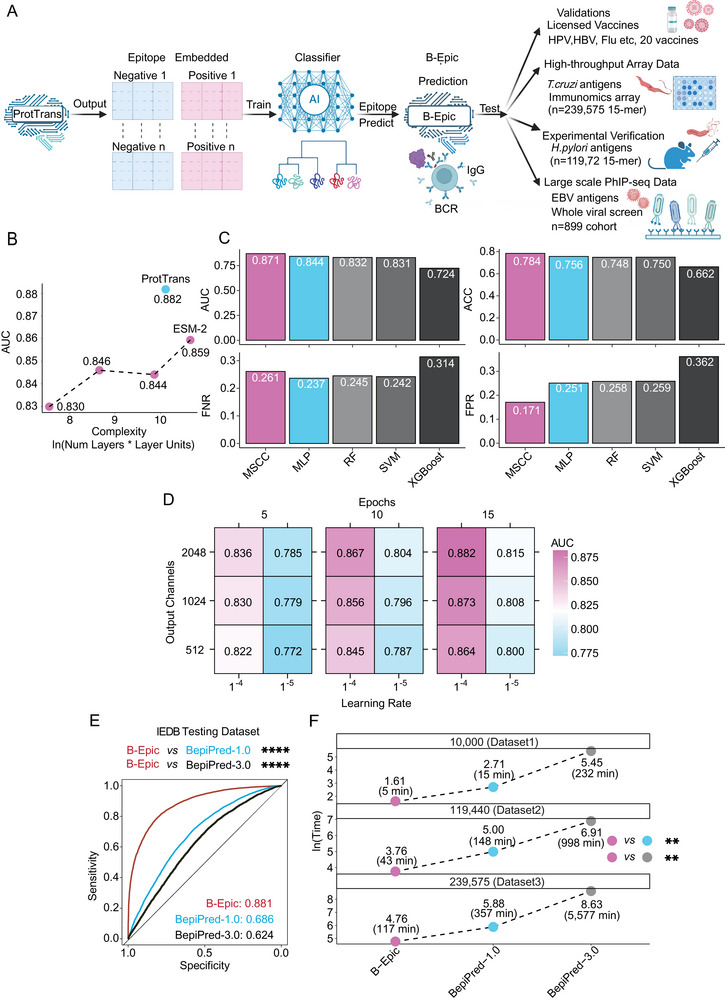
Development of B‐Epic: a Transformer‐based Approach for BCE Prediction. A) The development, validation, and application of B‐Epic were shown in the overview. B‐Epic leveraged the Transformer to extract semantic features of AA sequences and classify BCEs based on MSCC. Overall, B‐Epic was tested across the IEDB testing dataset, the peptidome of *Trypanosoma cruzi (* *T. cruzi)*, and licensed vaccine targets. The applications of B‐Epic included the *de novo* construction of a vaccine candidate library for *H. pylori* and the identification of pan‐immunoreactive peptides for EBV. B) A comparative performance assessment between two natural language models was presented, focusing on ESM‐2 (with varying complexity defined by Units*Numbers) and ProtTrans. C) A comparative performance assessment (MSCC and the other 4 machine learning approaches) was presented. Five classifiers were evaluated by AUC, ACC, FPR, and FNR. SVM, XGBoost, RF, and MLP preprocessed AA embeddings by converting them to sequence embeddings (mean pooling), whereas MSCC enables classification directly using AA embeddings. D) Hyperparameter optimization of MSCC was shown in the heatmap. The hyperparameter optimization process involved comparing AUC (0.772–0.875) across different learning rates (10^−4^, 10^−5^), output channels (512, 1024, 2048), and training epochs (5, 10, 15). The color gradient from blue to pink indicates increasing AUC. E) A comparative analysis of B‐Epic against BepiPred‐1.0 and BepiPred‐3.0 in the IEDB testing dataset was presented. F) It was presented that computational efficiency was compared across three increasingly large datasets containing 10 000, 119 440, and 239 575 samples, respectively. The computational time was processed using a natural logarithm (ln) transformation and used for subsequent statistical analyses. Statistical significance was denoted as follows: ^*^ *p* < 0.05; ^**^ *p* < 0.01; ^***^ *p* < 0.001; ^****^ *p* < 0.0001; ns (not significant). The significance level (α) was set at 0.05. Statistical analyses were performed using the two‐tailed Delong test (Figure 1E) and the paired two‐tailed t‐test (Figure 1F).

B‐Epic, a novel computational pipeline for predicting BCEs, leverages advanced natural language processing techniques. The training pipeline begins with length normalization of peptides from IEDB, encompassing 59 720 balanced peptides with corresponding positive or negative B cell activation experimental results. These sequences were transformed into AA embeddings via ProtTrans, which were then used for classifier training (**Figure** **1**A; Figure S1A,B, Supporting Information). The optimized B‐Epic demonstrated robust performance across diverse validations and showed practical utility in the design of immunological products for clinically relevant pathogens, including *H. pylori* and EBV (Figure 1A).

The classifier optimization process involved two key phases: comparison of different architectures and model parameter tuning (Figure S1C, Supporting Information). A key precondition for B‐Epic's construction was determining a suitable Transformer to extract features for BCE prediction. Both ESM‐2 and ProtTrans could extract features from AA sequences. In comparison, ProtTrans exhibited a higher AUC than ESM‐2 with comparable complexity, suggesting that ProtTrans was a reasonable model for BCE prediction (Figure 1B; Table S3, Supporting Information). Subsequently, the multi‐scale convolutional classifier (MSCC) achieved superior performance metrics compared to other classifiers, based on its high AUC and accuracy (ACC). Notably, MSCC exhibited the lowest false positive rate (FPR) while maintaining an acceptable false negative rate (FNR), which is a crucial advantage for reducing redundancy in downstream experimental validation (Figure 1C).

Through hyperparameter optimization within the defined parameter space, we determined that optimal performance was achieved with 2048 convolutional layer output channels, a batch size of 15, and a learning rate of 1e^−04^ (Figure 1D). Notably, kernel number and kernel size were another two other crucial parameters of the MSCC. The AUCs of MSCC with 3 kernels (sizes 2, 4, 8) were higher than those of MSCCs with 2 kernels (sizes 2, 4; 2, 8; 4, 8) and 1 kernel (sizes 2; 4; 8; Figure S1D, Supporting Information), though the variation was less pronounced compared to the effect of changing kernel sizes. The fluctuations in AUC for MSCCs with the same kernel number but different sizes emphasize the sensitivity of MSCCs to kernel size (Figure S1D, Supporting Information). Within the defined parameter space, the AUC gradually improved as the kernel size increased from 2 to 32 (maximum input peptide length was 32; Figure S1E, Supporting Information). Hence, within the defined parameter space, appropriately increasing the number or size of kernels benefited the enhancement of MSCC accuracy. All AUCs of MSCCs with different kernel numbers and sizes are provided in the supplementary materials (Table S4, Supporting Information).

Five‐fold cross‐validation yielded a median AUC of 0.884 (Figure S1F, Supporting Information), demonstrating B‐Epic's strong performance on the IEDB testing dataset. Comparative analyses revealed that B‐Epic significantly outperformed existing tools, achieving an AUC of 0.882 (95% CI: 0.877–0.885) compared to BepiPred‐1.0 (AUC = 0.686, 95% CI: 0.68–0.693) and BepiPred‐3.0 (AUC = 0.624, 95% CI: 0.617–0.631; Figure 1E; Table S5, Supporting Information). Importantly, B‐Epic maintained a significantly lower FPR (Figure S2A–D, Supporting Information), which is crucial for improving experimental success rate. The computational efficiency of B‐Epic surpassed that of both BepiPred‐1.0 and BepiPred‐3.0, with notably faster processing times for large‐scale peptide analyses (Figure 1F). B‐Epic's streamlined architecture makes it particularly well‐suited for high‐throughput applications in RV.

All in all, compared to BepiPred‐1.0 and BepiPred‐3.0 with commonly used, B‐Epic displayed higher accuracy and efficiency on the IEDB testing dataset, though its robustness requires further testing.

### 2.2 B‐Epic Screened Out the Targets of Licensed Vaccines from Random Sequences

**Figure 2:**
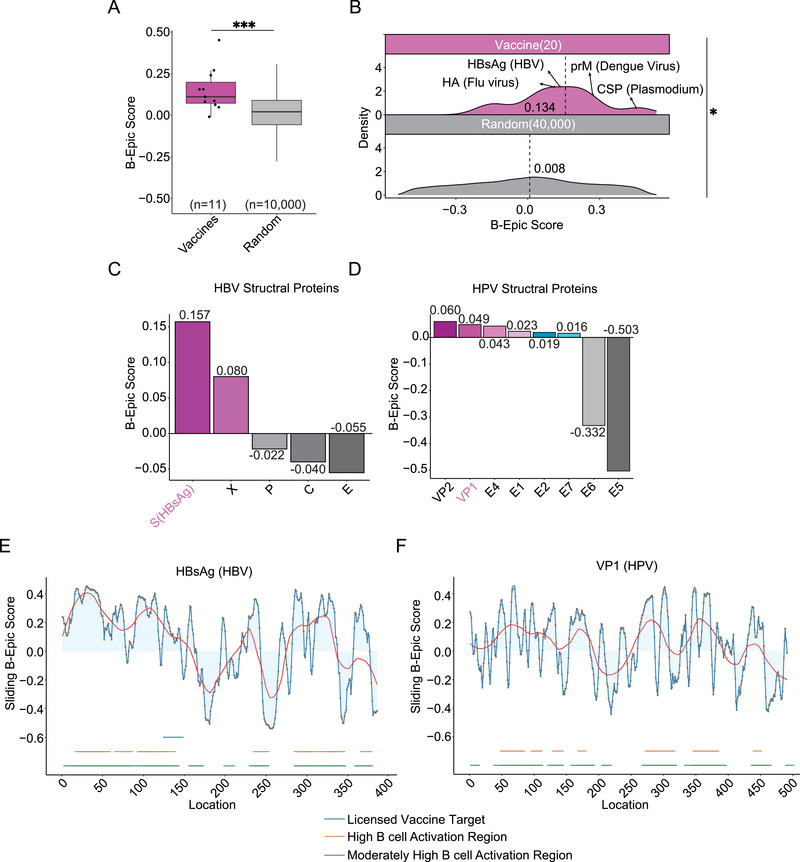
B‐Epic Screened out the Targets of Licensed Vaccines from Random Sequences. A) A comparative analysis of B‐Epic Score (the median ± IQR) between licensed vaccine protein targets (*n* = 11) and random protein controls (*n* = 10 000) was presented. B) Comparative analysis of B‐Epic Score encompassing 20 vaccine targets (proteins and peptides, with peptides ranging in length from 6 to 57 AAs) versus random sequence controls (*n* = 40 000) was presented, featuring key vaccine targets: HBsAg (HBV), CSP (Plasmodium), prM (dengue virus), and HA (influenza virus). C,D) Comparisons of B‐Epic Score for HBsAg (C; pink) and VP1 (D; pink) against their respective viral structural proteins (from their host species) were presented. E,F) Distributions of Sliding B‐Epic Score for HBsAg (E) and VP1 (F) were shown. Color segments were indicated as follows: known vaccine sequences (blue), high B cell activation regions (orange; B‐Epic Score > 0.35, Sliding B‐Epic Score > 0.25), and moderately high B cell activation regions (green; B‐Epic Score > 0.25, Sliding B‐Epic Score > 0.15). The red curve represented the LOESS trend based on Sliding B‐Epic Score. Statistical significance was denoted as follows: ^*^ *p* < 0.05; ^**^ *p* < 0.01; ^***^ *p* < 0.001; ^****^ *p* < 0.0001; ns (not significant). The significance level (α) was set at 0.05. Statistical analyses were performed using the two‐tailed Mann‐Whitney U test (Figure 2A,B).

To further validate B‐Epic's predictive capabilities, we systematically evaluated 11 licensed protein vaccine targets, including those against Human Papillomavirus (HPV), Hepatitis B Virus (HBV), and influenza virus. These clinically validated targets demonstrated significantly elevated B‐Epic Score compared to random proteins (**Figure** **2**A). We extended this analysis to 9 additional peptide vaccine targets (length 6–57 AAs), which similarly exhibited markedly higher B‐Epic Score than random sequences (Figure 2B), demonstrating B‐Epic's capacity to identify vaccine targets. Detailed information for the 20 vaccine targets is provided in the supplementary materials (Table S6, Supporting Information).

Taking vaccine targets Hepatitis B surface antigen (HBsAg; S) and Virus Protein 1 (VP1) as examples, B‐Epic was utilized to evaluate the potential of these two proteins for B cell activation. Notably, HBsAg achieved the highest B‐Epic Score among all structural proteins (X, P, C, E; Figure 2C). This predictive capability was further validated in HPV, where VP1 showed a high B‐Epic Score compared to most structural proteins (E1‐7; Figure 2D). As another structural protein of HPV with a high B‐Epic Score, VP2 has also been demonstrated to induce an IgG response by Shuai Shao.^[^ ^28^ ^]^ These horizontal comparisons demonstrated the capacity of B‐Epic to screen vaccine targets.

Detailed structural analysis of HBsAg and VP1 revealed extensive peptides with high and moderately high B‐Epic Score, indicating multiple potential B‐cell activating regions in these two proteins. Importantly, a licensed vaccine target within HBsAg (residues 124–149) showed concordance with high B‐Epic Score regions (Figure 2E,F). These detailed analyses explained the principle of B‐Epic to screen out HBsAg and VP1.

Collectively, B‐Epic is a powerful tool for identifying vaccine targets, though its predictive capacity requires further validation in pathogen peptidomes.

### 2.3 B‐Epic Identifies Immunoreactive Peptides from Peptidome of *T. cruzi*

**Figure 3:**
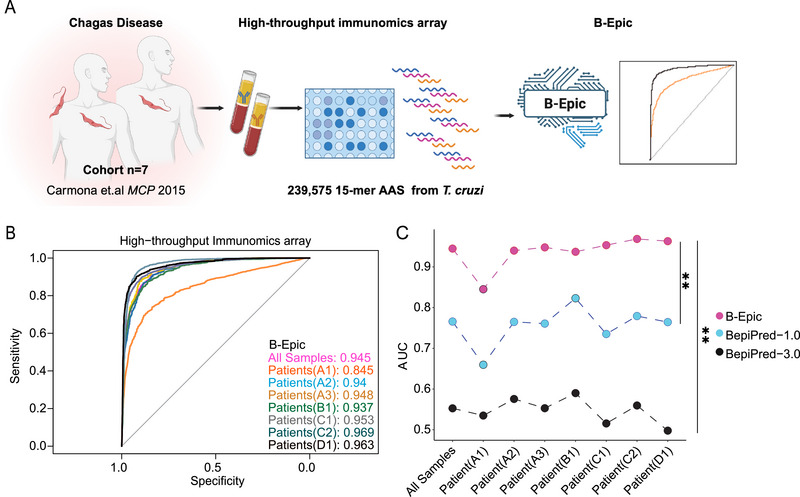
B‐Epic Identifies Immunoreactive Peptides from the Peptidome of *T. cruzi*. A) A schematic depicted the detection of immunoreactivity of *T. cruzi* peptidome (239575 peptides) from 7 sera of patients with Chagas disease via ELISA chips, as described in Santiago J. Carmona's article. B) The experimental results were binarized using reactogenicity thresholds of 3 (np‐neg; multiple samples) and 7 (np‐neg; per sample), as described in Santiago J. Carmona's article. AUCs of B‐Epic were calculated using the B‐Epic Score and binary results from the 7 ELISA chips. C) A comparative analysis was conducted to present the AUCs of B‐Epic, BepiPred‐1.0, and BepiPred‐3.0 in the peptidome of *T. cruzi* across 7 ESLIA chips. Statistical significance was denoted as follows: ^*^ *p* < 0.05; ^**^ *p* < 0.01; ^***^ *p* < 0.001; ^****^ *p* < 0.0001; ns (not significant). The significance level (α) was set at 0.05. Statistical analyses were performed using the two‐tailed Mann‐Whitney U test (Figure 3C).

Accurate identification of immunoreactive peptides from the peptidome is essential for BCE prediction tools. This predictive ability of B‐Epic was tested on the peptidome comprising 239 575 15‐mer peptides derived from 457 proteins of *T. cruzi*, the causative agent of Chagas disease, which induces severe cardiac and digestive complications. The specific antibody levels of these peptides, which reflect immunogenicity, were measured using 7 sera from patients with Chagas disease via ELISA chip technology (**Figure** **3**A). High accuracy in this large‐scale peptidome, which served as an ideal benchmark, would significantly boost confidence in the BCE prediction tools. B‐Epic achieved an average AUC of 0.936 in the peptidome of *T. cruzi* across 7 ELISA chips, demonstrating its remarkably high accuracy in large‐scale peptidome (minimum AUC = 0.845; Figure 3B). Notably, B‐Epic significantly outperformed BepiPred‐1.0 and BepiPred‐3.0 in the peptidome of *T. cruzi* across all 7 ELISA chips, showcasing its high superiority (Figure 3C; Figure S3A–C and Table S7, Supporting Information).

These results demonstrated B‐Epic's exceptional capability to discover immunoreactive peptides from large‐scale peptidomes, revealing its potential to accelerate the *de novo* development of vaccines. The robustness of B‐Epic was validated using the three aforementioned testing datasets, and based on these validations, the next step is to develop its applications.

### 2.4 *De Novo* Development of *H. pylori* Potential Vaccine Candidate Library with Experimental Validation

**Figure 4:**
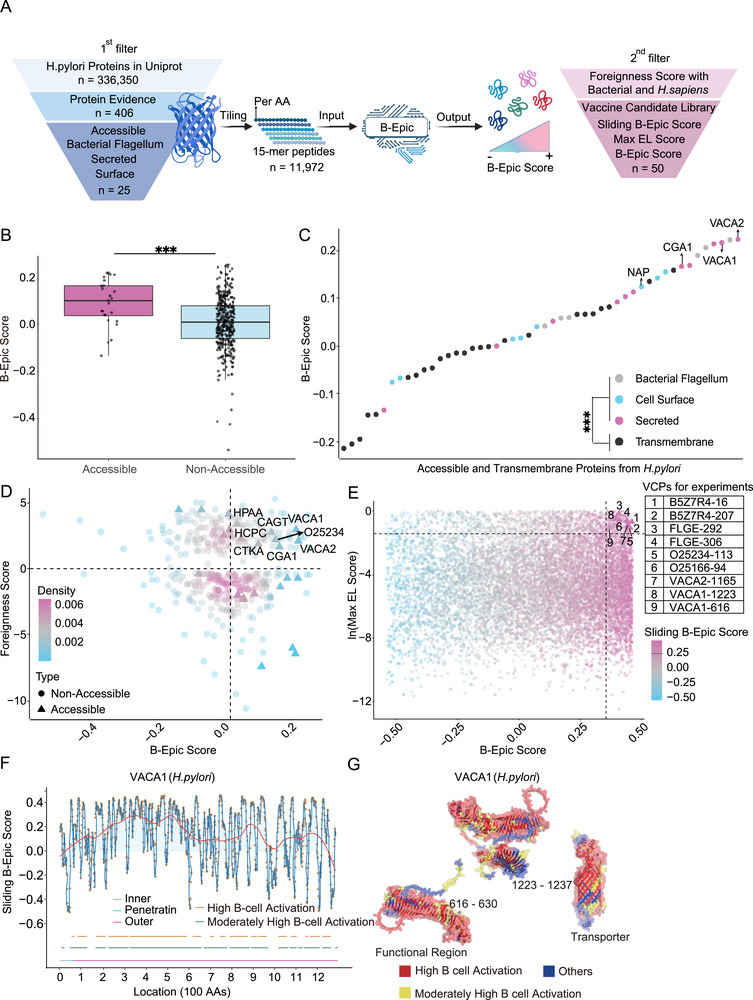
De *Novo* Development of *H. pylori* Potential Vaccine Candidate Library with Experimental Validation. A) The construction of a vaccine candidate library of *H. pylori* was shown. From 336 350 *H. pylori* sequences in UniProt, 25 accessible proteins (as defined in the Methods section) were split into 15‐mer peptides using a sliding window with a step size of 1 AA. Overall, 11972 15‐mer peptides were input into B‐Epic, with 50 of these peptides were ultimately included in the vaccine candidate library. B) A comparison of B‐Epic Score between accessible and non‐accessible *H. pylori* proteins was presented (the median ± IQR). C) B‐Epic Score rankings of 25 accessible proteins and 24 transmembrane proteins were shown. Gray, turquoise, pink, and black represented bacterial flagellum, cell surface, secreted, and transmembrane proteins, respectively. NAP, CGA1, VACA1, and VACA2 were potential vaccine targets with experimental evidence of B‐cell activation. D) The protein‐level vaccine library was constructed from the accessible proteins. Thresholds were set as: B‐Epic Score > 0.02 (median B‐Epic Score of random proteins) and Foreignness Score > 0 (negative Bit Score of DIAMOND). Triangles represented accessible proteins, and circles represented non‐accessible proteins. E) Overall, 11 972 peptides were generated from 25 accessible proteins. For these peptides, the x‐ and y‐axes represented B‐Epic Score and ln(Max EL Score), with thresholds of 0.35 and ln(0.25), respectively. The color gradient from blue to pink indicated the Sliding B‐Epic Score with a threshold of 0.25. In addition, a Foreignness Score > 0 was considered during construction of the peptide‐level vaccine library, but wasn't displayed in this chart. The 9 VCPs for subsequent experiments were highlighted in the table (right). F) The distribution of Sliding B‐Epic Score for VACA1 was exhibited. Orange, green, and red lines represented high B cell activation regions, moderately high B cell activation regions, and the LOESS trend based on Sliding B‐Epic Score, respectively. Turquoise, pink, and gray lines represented penetrating, outer membrane, and inner membrane, predicted using TMHMM v2.0c. G) The surface and secondary structure of VACA1 were shown. This structure contained functional regions and transporters. VACA1‐1223 and VACA1‐616 (for subsequent experiments) were highlighted by reduced transparency on the VACA1 surface (rendered with high overall transparency). Surface rendering with high transparency distinguished between high B cell activation regions (red), moderately high B cell activation regions (orange), and remaining regions (blue). Statistical significance was denoted as follows: ^*^ *p* < 0.05; ^**^ *p* < 0.01; ^***^ *p* < 0.001; ^****^ *p* < 0.0001; ns (not significant). The significance level (α) was set at 0.05. Statistical analyses were performed using the two‐tailed Mann‐Whitney U test (Figure 4B,C).

The gastric pathogen *H. pylori*, classified as a Group I carcinogen, presents a significant challenge in GC prevention due to its increasing antimicrobial resistance and extensive strain diversity.^[^ ^29^ ^]^ Given the limitations of conventional antibiotic therapies, we utilized B‐Epic to *de novo* identify vaccine targets against *H. pylori* infections.^[^ ^30^, ^31^, ^32^ ^]^ BCEs of *H. pylori* with experimental evidence (Table S6, Supporting Information) had higher B‐Epic Score than random sequences, supporting the potential of B‐Epic in the development of vaccines against *H. pylori* (Figure S4A,B, Supporting Information). To showcase B‐Epic's practical utility, we *de novo* established a vaccine candidate library using 406 proteins (“Evidence at protein level”; PE1) of *H. pylori* from UniProt (**Figure** **4**A).

Accessible proteins, including those located on bacterial flagella, cell surface, and secreted proteins, are most likely to engage with host immune responses.^[^ ^33^, ^34^ ^]^ Overall, accessible proteins exhibited higher B‐Epic Score compared to transmembrane and non‐accessible proteins (Figure 4B,C). Notably, among accessible proteins, NAP, VACA1/2, and CGA1, which were involved in previous clinical trials,^[^ ^35^ ^]^ had high B‐Epic Score (Figure 4C). These findings not only further demonstrated the potential of B‐Epic for application in vaccine design of *H. pylori*, but also provided justification for the rationale of constructing a vaccine candidate library based on 25 accessible proteins.

Our vaccine candidate libraries were constructed at both the protein and peptide levels. To minimize potential side effects such as autoimmunity, foreignness was evaluated based on the dissimilarity of *H. pylori* sequences compared to those of *H. sapiens* and other prokaryotes. Eventually, 8 accessible proteins with B‐Epic Score higher than the median of 10 000 random proteins (0.02), and significant foreignness (Foreignness Score > 0) were included in the protein‐level library (Figure 4D).

To establish the peptide‐level library, 11 972 15‐mer peptides were generated from the 25 accessible proteins using a sliding window with a step size of 1 AA (Table S8, Supporting Information). In addition to the Foreignness Score and B‐Epic Score, we also considered the Sliding B‐Epic Score and Max EL Score (NetMHCIIpan). The Sliding B‐Epic Score was included to mitigate the impact of outliers, while the Max EL Score was considered due to the synergistic effect of simultaneous activation of both B cells and T cells on antibody production.^[^ ^36^, ^37^, ^38^ ^]^ This rigorous filtering process (B‐Epic Score > 0.35; Sliding B‐Epic Score > 0.25; Foreignness Score > 0; Max EL Score > 0.25) yielded 50 high‐confidence vaccine candidate peptides (VCPs), 9 of which were validated in subsequent experiments (Figure 4E).

As a classical example, VACA1 and its two 15‐mer peptides in the vaccine candidate libraries were used to briefly explain B‐Epic's operating principle. A high overall B‐Epic Score indicated multiple potential B‐cell activating regions on VACA1 (Figure 4F). Within VACA1, we identified two promising peptides: VACA1‐616 and VACA1‐1223, which were both outside the toxic region (1–494). These two peptides are strategically located in β‐turn loops, structures typically associated with BCE accessibility.^[^ ^39^ ^]^ VACA1‐616 resides in a functional domain critical for gastric mucosal disruption through osmotic pressure modulation, while VACA1‐1223 is positioned in the autotransporter region responsible for functional domain translocation (Figure 4G).^[^ ^40^ ^]^

**Figure 5:**
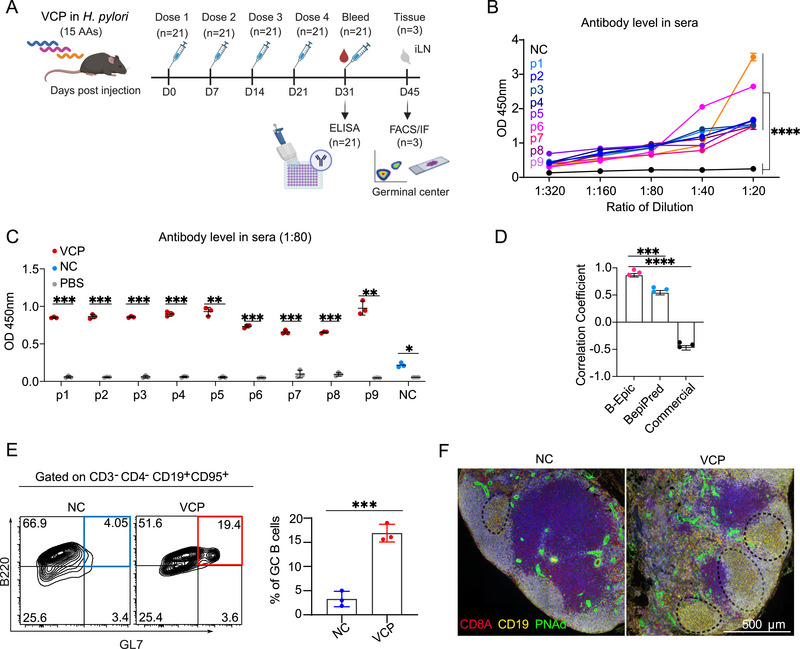
Immunization with Predicted *H. pylori* BCEs Elicits Robust Antibody Responses in Mice. A) A schematic of the immunization protocol was presented. C57BL/6 mice (*n* = 21) received subcutaneous injections of VCPs formulated with CpG adjuvant. B) Dose‐dependent ELISA detected the expression of specific antibodies against VCPs (*n* = 9; 3 replicates per VCP) and NC (*n* = 1; 3 replicates per NC) in mouse sera, demonstrating the specificity of the immune response across multiple serum dilutions. C) Quantification of specific IgG responses at a 1:80 dilution was shown. Mice immunized with VCPs or NC showed significantly elevated antibody titers compared to PBS controls. D) Correlation analysis between predictive results and ELISA results was presented. E) Flow cytometric quantification of GC B cells following peptide immunization. Blue and red squares represented distinct GC B cell populations. F) Representative immunofluorescence images of lymph nodes showed a lymph node marker (green), along with staining of B cell (yellow) and T cell (red) populations. Points in statistical charts (C,E) represented individual mice. Data were presented as the mean ± SD (B‐E; some small SDs not visually distinguishable). Statistical significance was denoted as follows: ^*^ *p* < 0.05; ^**^ *p* < 0.01; ^***^ *p* < 0.001; ^****^ *p* < 0.0001; ns (not significant). The significance level (α) was set at 0.05. Statistical analyses were performed using the two‐tailed two‐way ANOVA (Figure 5B) and two‐tailed t‐test (Figure 5C–E).

Nine VCPs in the library (Figure 4E; Table S8, Supporting Information) were synthesized and KLH‐conjugated to undergo comprehensive immunization studies in mice. VCPs with B‐Epic Score ranging from 0.35 to 0.46 were tested against NC (B‐Epic Score: −0.54; *H. pylori*). Following four immunization rounds, by ELISA, OD_450nm_ absorbance for all VCPs was significantly higher relative to the NC (*P* < 0.0001), though detectable antibody reactivity was observed in NC (*P* < 0.05; **Figure** **5**A–C; Figure S5A and Table S9, Supporting Information). Moreover, compared to BepiPred tools and a commercial software (name not disclosed for commercial reasons) for BCE prediction, ELISA results exhibited stronger correlation with B‐Epic Score (Figure 5D). Flow cytometry and immunofluorescence analyses of inguinal lymph nodes demonstrated significant GC B cell formation in VCP‐immunized mice, although T follicular helper cell populations remained unchanged (Figure 5E,F; Figure S6A,B, Supporting Information). These results provide strong experimental validation of B‐Epic's predictive accuracy.

In summary, we have *de novo* established comprehensive *H. pylori* vaccine candidate libraries at both the peptide and protein levels using B‐Epic, significantly improving the efficiency of vaccine design against *H. pylori*. The VCPs with experimental evidence provided a solid foundation for future *H. pylori* vaccine development efforts.

### 2.5 B‐Epic Exhibited Exceptional Performance on Identifying Pan‐Immunoreactive Peptides of EBV in Large Clinical Cohort

EBV, a pervasive oncogenic herpesvirus, orchestrates complex immune modulation mechanisms that contribute to various human malignancies and autoimmune disorders. Different EBV strains exhibit distinct pathogenic profiles; for instance, B95‐8 and AG876 are associated with lymphoma development, while GD‐1 shows a strong correlation with NPC.^[^ ^41^ ^]^ Despite exposure to multiple strains throughout life, most individuals remain asymptomatic, likely due to effective protective antibody responses.^[^ ^42^ ^]^ Intriguingly, while over 90% of the global population is infected with EBV, the majority never develop EBV‐related diseases. This widespread viral prevalence, combined with diverse antigenic profiles in healthy individuals, presents an unprecedented opportunity to identify broadly protective vaccine candidates.^[^ ^43^, ^44^ ^]^ We leveraged phage‐display libraries as a robust experimental pipeline for mapping BCEs, where competitive immuno‐screening uncovered BCE signatures through systematic sequence alignment analysis.^[^ ^45^ ^]^

**Figure 6:**
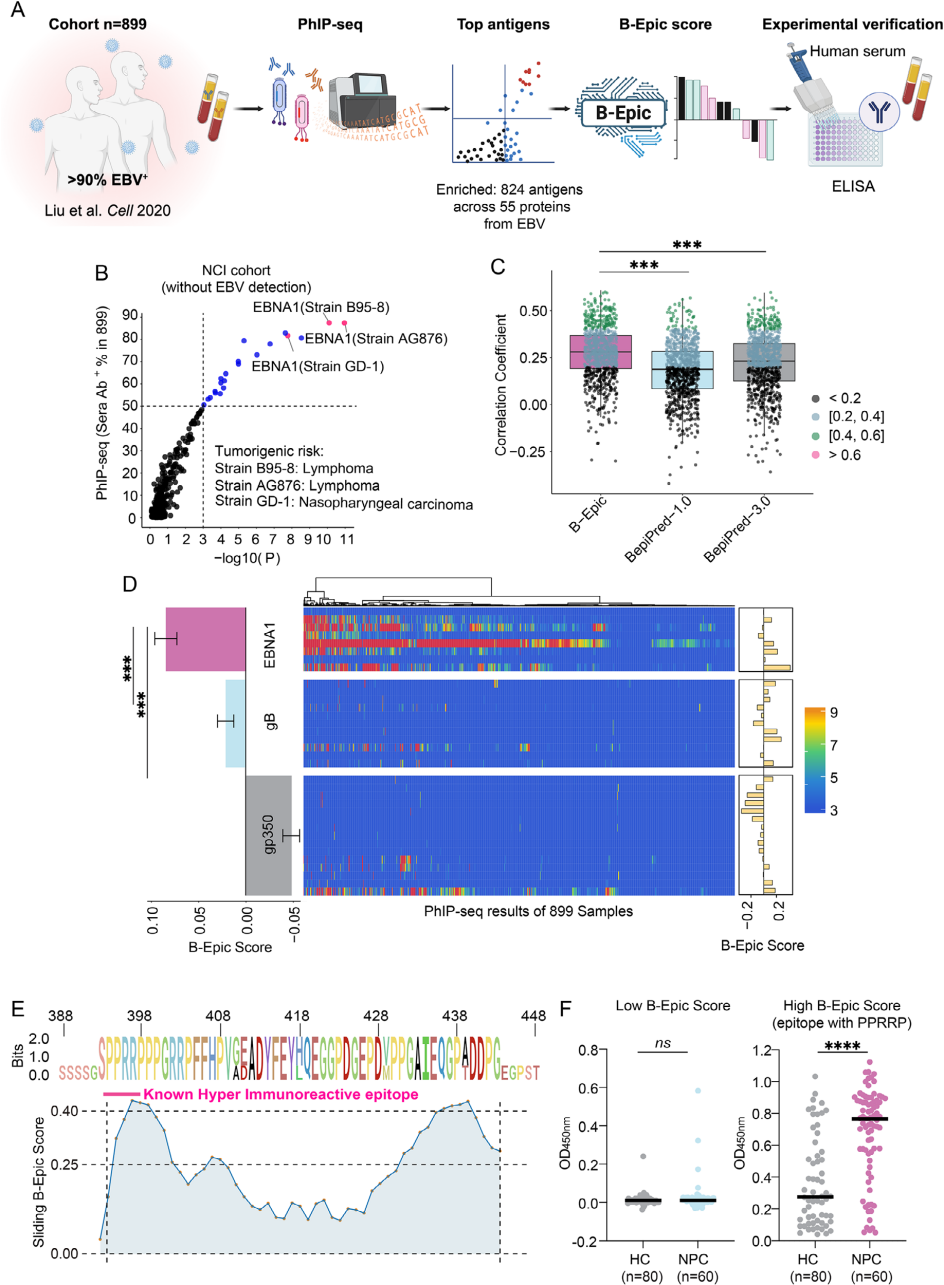
B‐Epic Exhibited Exceptional Performance on Identifying Pan‐Immunoreactive Peptides of EBV in Large Clinical Cohort. A) A schematic of B‐Epic's application against EBV was presented. The correlation between PhIP‐seq and B‐Epic in a large clinical cohort across 899 sera was analyzed, and specific antibody levels against EBV peptides with high B‐Epic scores were detected via ELISA in another large cohort. B) The y‐axis represented serological positivity rates across 899 sera based on PhIP‐seq enrichment. Blue dots indicated antigens meeting significance thresholds (> 50% prevalence, PhIP‐seq *P* < 0.001). Pink dots highlighted the high pan‐immunogenicity of EBNA1 across three EBV strains. C) The chart presented a comparative analysis of Spearman correlation coefficients between PhIP‐seq enrichment (34 peptides) and predictions from B‐Epic, BepiPred‐1.0, and BepiPred‐3.0 across 899 sera. Different intervals of correlation coefficient were colored as follows: grey (< 0.2), blue (0.2–0.4), green (0.4–0.6), and pink (> 0.6). D) Integrated visualization was generated to present the B‐Epic Score for EBNA1, gp350, and gB from strain AG876 (left), PhIP‐seq enrichment patterns across 899 sera (center heatmap), and corresponding B‐Epic Score of sequences in the PhIP‐seq assay (right). E) Distribution of Sliding B‐Epic Score for EBNA1 393–448 across three EBV strains was shown. The pink line represented the known highly immunoreactive epitope “PPRRP”. F) ELISA detected levels of specific antibodies against EBNA1 15‐mer peptides with high/low B‐Epic Score in NPC patients (*n* = 80) and healthy controls (*n* = 60). Statistical significance was denoted as follows: ^*^ *P* < 0.05; ^**^ *P* < 0.01; ^***^ *P* < 0.001; ^****^ *P* < 0.0001; ns (not significant). The significance level (α) was set at 0.05. Statistical analyses were performed using the two‐tailed Mann‐Whitney U test (Figure 6C) and two‐tailed t test (Figure 6F).

A large PhIP‐seq clinical cohort^[^ ^45^ ^]^ comprising virome‐wide serological profiles from 899 individuals with demographic information (Table S10, Supporting Information) was used to evaluate B‐Epic's capacity to identify pan‐immunoreactive peptides for EBV immunological product development (**Figure** **6**A). Our analysis of the complete EBV proteome revealed 824 distinct PhIP‐seq enrichments of peptides across 55 proteins from three major tumorigenic strains (B95‐8, AG876, and GD‐1). Remarkably, EBNA1 was the predominant pan‐immunoreactive protein, with notably high serological positivity rates (> 85%, *P* < 0.001) across 899 human sera in this large PhIP‐seq clinical cohort. Moreover, it ranked as the pan‐immunodominant antigen in the three tumorigenic EBV strains (AG876, Akata, and B95‐8), demonstrating its high pan‐immunoreactive conservation. The pan‐immunoreactive conservation of EBNA1 across three EBV strains in this large clinical cohort provides critical support for validating B‐Epic's capability to predict pan‐immunoreactivity. (Figure 6B).

Overall, comparative analysis demonstrated that B‐Epic Score exhibited a significantly higher correlation with PhIP‐seq enrichment across 899 sera compared to BepiPred‐1.0 and BepiPred‐3.0 (Figure 6C; Figure S7A, Supporting Information). In detail, an elevated B‐Epic Score of peptides predicted enhanced PhIP‐seq enrichment (Figure 6D; Figure S7B,C, Supporting Information). These results underscore that B‐Epic can effectively predict PhIP‐seq enrichment in the large clinical cohort, indicating its capacity to forecast pan‐immunoreactivity.

The immunodominance of EBNA1 has been well‐established, with previous studies demonstrating its potential in inducing B cell activation compared to gB and gp350, suppressing EBV latency, and inhibiting tumor growth in preclinical models.^[^ ^5^, ^46^ ^]^ This was further supported by its higher B‐Epic Score compared to gB and gp350, with most EBNA1 peptides displaying high PhIP‐seq enrichment (Figure 6D; Figure S7B,C, Supporting Information). Conversely, gp350 (B‐Epic Score: −0.043) has been reported to fail to demonstrate efficacy in protecting vaccinated populations from EBV infection (Figure 6D).^[^ ^47^, ^48^ ^]^ With the highest PhIP‐seq enrichment, the EBNA1 peptide (393–448) had a median B‐Epic Score of 0.263 across three EBV strains. In addition, the known highly immunoreactive EBNA1 epitope “PPRRP” which was confirmed by previous findings, also had a median B‐Epic Score of 0.095 across three EBV strains (Figure 6E).^[^ ^49^ ^]^ All in all, B‐Epic could reflect the immunoreactive conservation of the EBNA1 peptide (393–448) and “PPRRP”.

Based on these results, a total of two EBNA1 peptides with high B‐Epic Score (Table S8, Supporting Information) were synthesized to test their pan‐immunoreactivity in another large clinical cohort containing sera from 80 NPC patients and 60 HCs. Peptides with high B‐Epic Score exhibited significantly higher OD_450nm_ absorbance in NPC patients than in HC. In contrast, peptides with low B‐Epic Score (Table S2, Supporting Information) showed insignificant OD_450nm_ absorbance differences between NPC patients and HC (Figure 6F; Figure S7D, Supporting Information). These results demonstrated that peptides with high B‐Epic Score had pan‐immunoreactivity, further validating the capacity of B‐Epic.

The pan‐immunoreactivity of peptides from EBV screened by B‐Epic was validated through rigorous experiments in a large clinical cohort, highlighting the potential of these peptides for vaccine development and patient stratification.

## 3 Discussion

Our study advances the field of computational BCE prediction, while highlighting critical areas for improvement. The development of B‐Epic demonstrates substantial progress in predicting BCEs, particularly its ability to perform high‐throughput analysis of the peptidome and even the proteome. This superiority proves especially valuable for the development of immunological products, such as vaccines, immunodiagnostics, and immunotherapeutics, where the identification of immunogenic targets is paramount. Detailed parameters of B‐Epic and guidelines for installation and usage are provided on GitHub (https://github.com/LiangJzzz/B‐Epic‐1.0.git).

In our study, we revealed both the capabilities and limitations of current computational approaches for BCE prediction. While traditional machine learning models such as RF, XGBoost, and SVM have shown promise, their prediction accuracy has historically been modest. Manually derived features, including subcellular location, surface accessibility, flexibility, and hydrophilicity, provide valuable structural insights but primarily indicate rough antibody‐accessible regions rather than definitive BCE locations. Unlike the Transformer, traditional machine learning models struggle to autonomously extract features and therefore, rely on manually derived features, which limits their accuracy. Traditional machine learning models, on the other hand, typically require complex preprocessing to handle the PPS of AA, whereas the Transformer can directly process AA sequences. In summary, these highlight the disadvantages of traditional machine learning models compared to the Transformer.

Additionally, MSCC exhibits clear advantages over traditional machine learning models. First, it is capable of directly processing the AA embedding matrices output by the Transformer, eliminating the need for lossy pooling typically required in traditional machine learning models. Second, MSCC utilizes multiple convolutions to capture both local and global features more effectively, enabling it to learn a broader range of features compared to traditional machine learning models. All in all, these highlight the advantages of MSCC over traditional machine learning models.

Previous studies showed that most computational methods struggle to achieve AUC values above 0.8^[^ ^22^, ^50^, ^51^ ^]^ or lack experimental validation,^[^ ^52^, ^53^ ^]^ indicating the nascent stage of the BCE prediction field. Notably, B‐Epic demonstrated improved performance, with high accuracy across several heterogeneous benchmarking datasets, suggesting substantial progress. Through rapid and accurate prediction, B‐Epic addresses the growing demand for precision medicine, thereby expanding the application of BCE identification beyond vaccines to encompass immunodiagnostic tools and immunotherapeutic antibodies.

In the context of *H. pylori* applications, the multiple epitope vaccine (MEV) is an immunological product suitable for development using B‐Epic, a process that requires a comprehensive and precise vaccine candidate library. The advantages of MEV include the stable, large‐scale production of short peptides via chemical synthesis; the ability to generate broader immune responses through multiple epitopes; and the potential to avoid homologous regions, thereby reducing side effects and autoimmune responses.^[^ ^54^ ^]^ During validation of immune response to *H. pylori* VCPs versus NC, administration of CpG adjuvant elicited robust immune responses. CpG is an adjuvant that stimulates the immune system via Toll‐like receptor 9 (TLR9), whose expression is upregulated in mice following *H. pylori* infection, with elevated levels primarily observed in macrophages and dendritic cells. Given the reported critical role of Toll‐like receptors in recognizing *H. pylori* during infection, the usa of CpG adjuvant can better mimic the pro‐inflammatory process triggered by TLR9‐mediated bacterial DNA recognition in human hosts.^[^ ^55^, ^56^ ^]^ Our results demonstrated the practical potential of combining synthetic peptides with CpG in future vaccine development. Furthermore, given the higher prevalence of *H. pylori* infection in males, male mice formed the majority of the cohort in our BCE validation trials.^[^ ^57^ ^]^

In addition, five challenges still persist with B‐Epic. First, B‐Epic, which was trained on linear epitopes, struggles to fully capture the complexity of conformational epitopes.^[^ ^58^ ^]^ Hence, predicting 3D epitopes using B‐Epic is out of scope at present. Second, post‐translational modifications are another core factor influencing the structure of BCEs. For highly glycosylated proteins, the accuracy of B‐Epic may be affected, a consideration users should be aware of. Notably, since B‐Epic was primarily trained on peptides of lengths 12–16, its high predictive accuracy is confined to this length range. The B‐Epic Score of long sequences exceeding 15 AAs were calculated by its derived 15‐mer peptides generated using a sliding window. Compared to a sliding window with a step size of 1, the mean square errors of windows with step sizes of 2–10 were 0.00039–0.0024 (Table S1, Supporting Information), exhibiting small variations in accuracy, while computational efficiency was significantly improved. Hence, it is recommended that users increase the sliding window size when screening BCEs in large datasets. Third, unlike thresholds set at the peptide level, FDR can be determined based on the distribution of sufficient negative samples. However, quality control at the protein level still requires a greater quantity of proteins (with corresponding experimental results of B‐cell activation) to establish reliable standards. Fourthly, B‐Epic's performance on pairs of unmutated/mutated sequences from the IEDB testing dataset remained stable (AUC = 0.943, Table S11, Supporting Information). B‐Epic tended to predict minimal differences for mutations, likely because most mutations in the IEDB training/testing dataset have a limited effect on B‐cell activation. However, for key mutations that have a significant effect on B cell activation (Table S11, Supporting Information), the AUC of B‐Epic significantly decreased (Figure S7E, Supporting Information), highlighting a substantial area that requires further refinement. Despite this, B‐Epic still exhibited a higher AUC (Figure S7E, Supporting Information) in terms of distinguishability (Figure S7F, Supporting Information) compared to BepiPred‐1.0 and BepiPred‐3.0. Fifthly, the testing data for RNA viruses is relatively limited compared to other pathogens, such as DNA viruses (EBV) and bacteria (*H. pylori and T. cruzi*). Although B‐Epic still demonstrates strong predictive capabilities with the available RNA virus data (Figure S7G; Table S12, Supporting Information), this limitation should be acknowledged.

Building upon these promising results, our work opens up several exciting avenues for expanding B‐Epic's capabilities. The robustness of B‐Epic, particularly in identifying BCEs that have been experimentally validated, suggests its potential for broader applications in vaccine development. Future developments could include integrating advanced deep learning architectures, expanding SCEptRE annotations, incorporating additional immunological parameters, and extending prediction capabilities to more pathogens.^[^ ^59^, ^60^, ^61^, ^62^, ^63^ ^]^ Notably, 3D features are also profoundly impactful for BCE prediction. Future developments of B‐Epic will focus on bridging the gap between sequence‐based and structural‐based prediction, enabling it to learn 3D features from a database of 3D epitopes, potentially revolutionizing computational antibody design and vaccine development. These enhancements would further strengthen B‐Epic's position as a powerful tool for efficient vaccine design.

## 4 Conclusion

B‐Epic was the first deep learning pipeline to apply the Transformer in high‐throughput and accurate prediction of BCEs, addressing urgent needs in the field of immunological product development. The exceptional capabilities of B‐Epic have been comprehensively demonstrated through three validation studies (IEDB testing dataset, *T. cruzi* peptidome, and licensed vaccine targets).

*H. pylori* and EBV are pathogens associated with GC and NPC, respectively, but there is a lack of preventive strategies for both. Based on its performance, B‐Epic has been applied in the development of immunological products. We *de novo* constructed a vaccine candidate library for *H. pylori*, and the VCPs were shown to activate B cells in our experiments. The construction of the vaccine candidate library was beneficial for the development of vaccines against *H. pylori*. In another application, B‐Epic could screen for pan‐immunoreactive peptides in a large clinical cohort (*n* = 899). Notably, these peptides also had higher immunoreactivity in NPC patients than HC (*n* = 140), implying they had the potential to serve as not only vaccines but also immunodiagnostics. The B‐Epic Score of peptides involved in this article is listed in the (Table S12, Supporting Information).

B‐Epic facilitates the clinical disease prevention efforts, such as enabling *de novo* vaccine development and the design of immunodiagnostics, ultimately alleviating the health burden caused by pathogens.

## 5 Experimental Section

### Hardware

The GPU model used was the A100 with 40 GB of VRAM. The CPU model used was the Intel(R) Xeon(R) Platinum 8338C, featuring 64 threads and a total memory of 503 GB. The A100 was used to maximize the computational efficiency of B‐Epic and BepiPred‐3.0, both of which support GPU acceleration.

### Database Resources and Data Collection

The BCEs from the Immune Epitope Database (IEDB) were utilized to train and test B‐Epic. The selection criteria for the training/testing dataset were as follows: exclusively linear epitopes, *Homo sapiens* (*H. sapiens*) as the host organism, and IgG‐specific responses. The experimental outcomes of B cell activation assays were used to categorize peptides as positive or negative (downloaded on September 10, 2024).

Santiago J. Carmona measured the expression of specific IgG against 239 575 peptides from 472 *Trypanosoma cruzi* (*T. cruzi*) proteins on enzyme‐linked immunosorbent assay (ELISA) chips with sera from seven Chagas disease patients (A1, A2, A3, B1, C1, C2, D1); this is a conventional and reliable methodology.^[^ ^16^ ^]^ The experimental results were binarized using thresholds of reactogenicity, which were 3 (np‐neg; multiple samples) and 7 (np‐neg; per sample). “np” denoted the ELISA signal in patients with Chagas disease, and “neg” denoted the ELISA signal in negative control (NC).^[^ ^16^ ^]^ In addition, the method for calculating the Sliding B‐Epic Score was also adapted from Santiago J. Carmona's study to reduce the impact of outliers.^[^ ^16^ ^]^

A total of 336 350 amino acid (AA) sequences of *Helicobacter pylori* (*H. pylori*) were retrieved from UniProt. The focus was on proteins with experimental validation (“Evidence at protein level”; PE1; *n* = 406). Accessible proteins (*n* = 25) were identified using cellular localization terms: “Secreted”, “Cell outer membrane”, “Cell surface”, “Lipid‐anchor”, and “Bacterial flagellum”. Transmembrane proteins (*n* = 24) were identified using the cellular localization term “Transmembrane” (data downloaded on November 6, 2024).

### Sampling Method

Undersampling was employed to balance the positive and negative samples. To avoid bias in B‐Epic's predictive performance due to peptide length, positive and negative samples were divided into subsets based on lengths ranging from 2 to 32 AAs. Within each length subset (2 to 32 AAs), the majority class was randomly undersampled without replacement to match the peptide count of the minority class.

### Construction of B‐Epic

Detailed parameters of B‐Epic and guidelines for installation and usage were provided on GitHub (https://github.com/LiangJzzz/B‐Epic‐1.0.git).

### Construction of B‐Epic—Feature Extraction Using ProtTrans

ProtTrans with 24 hidden layers (1024 units per layer) and 32 attention heads, pre‐trained on protein sequences from UniRef50 was employed, to extract semantic features from protein sequences. ProtTrans was trained with 15% AA masking, utilizing a feed‐forward network of 16 384 units. Training was performed with a batch size of 512, a learning rate of 0.1, and gradient accumulation steps of 8. The Adam optimizer was employed for optimization over 991 000 training steps, resulting in a model with 3 billion parameters.^[^ ^24^ ^]^

### Construction of B‐Epic—Protein sequence preprocessing and embedding generation

Individual AA sequences were tokenized with space separation (e.g., “D E K R … D P A S”) and processed through the model's hidden layers. The semantic embedding matrix was extracted from the final hidden layer. The shape of the hidden layer was as follows:

(1) $$\left[Semantic\;Features,\;Sequence\;Length\right]$$

### Construction of B‐Epic—Development of Semantic Classifiers

For classifier training, peptides were converted into AAs embedding matrices. Due to the limitations of traditional machine learning approaches (Multi‐Layer Perceptron (MLP), Random Forest (RF), Support Vector Machine (SVM), and XGBoost) in directly processing matrices, mean pooling was implemented to transform the AAs embedding matrices into a single sequence matrix. These classifiers were implemented using default parameters from the scikit‐learn package.

### Construction of B‐Epic—Multiple Scale Convolutional Classifier (MSCC) Architecture

MSCC was independently developed to directly process AA embedding matrices without mean pooling. The architecture comprised 3 convolutional kernels, each extracting 2048 features. The subsequent fully connected layer with 7168 units incorporated both the raw output of ProtTrans and newly extracted convolutional features for binary classification. Training was conducted using the Adam optimizer (learning rate = 1e^−04^) over 15 epochs with ReLU activation. MSCC incorporated 3 convolutional kernels with sizes of 2, 4, and 8.

### Construction of B‐Epic—Calculation of B‐Epic Score and Its Derivative Score

Constructing a scoring system based on B‐Epic facilitated a more intuitive and interpretable BCE prediction.

For quantitative assessment, the following scoring methodology was implemented for each target peptide (*Ta*) to generate the classification result:

(2) $$\mathrm{Classification}=\left[z_{Ta,p},z_{Ta,n}\right]$$

The Raw Score was defined as the probability of positive peptide classification, calculated by the Softmax function providing a continuous value between 0 and 1, reflecting the likelihood of the target peptide being classified as positive:

(3) $$Raw\;Score=\left(\frac{z_{Ta,p}}{z_{Ta,p}+z_{Ta,n}}\right)$$

where *z*_*Ta*,*p*_ and *z*_*Ta*,*n*_ represent the logits corresponding to the positive and negative classes, respectively, in this binary classification framework.

The B‐Epic Score was normalized by comparing the target peptide's Raw Score against the median Raw Score of 10 000 length‐matched random peptides. The B‐Epic Score represents the standardized propensity for B cell activation, with positive values indicating enhanced immunogenic potential relative to the random peptide baseline. The B‐Epic Score for each target peptide was calculated as follows:

(4) $$s_{Ra}=\left\{Raw\;Score_{Ra,1},Raw\;Score_{Ra,2},\text{…},Raw\;Score_{Ra,10000}\right\}$$

*s_Ra_* denotes the set of Raw Scores for the 10 000 random peptides (match in length to target peptide *Ta*), and *Raw* *Score*_*Ra*,*i*_ represents the Raw Score of the *i^th^* random peptide in the *s_Ra_*.

The B‐Epic Score for the target peptide *Ta* was computed as follows:

(5) $$B\;Epic\;Score_{Ta}=Raw\;Score_{Ta}-median\left(s_{Ra}\right)$$

where *B* *Epic* *Score_Ta_* is normalized by subtracting the median of *s_Ra_* from the *Raw* *Score_Ta_* (the Raw Score of the target peptide *Ta*).

For sequences exceeding 15 AAs, a sliding window approach was implemented to split the sequence into a set of 15‐mer peptides:

(6) $$s_{Ta}=\left\{B\;Epic\;Score_{Ta,1},B\;Epic\;Score_{Ta,2},\text{…},B\;Epic\;Score_{Ta,n-14}\right\}$$

where *s_Ta_* represents the set of B‐Epic Score for all 15‐mer peptides derived from target sequence *Ta*, and *n* denotes the length of target peptide *Ta*.

The B‐Epic Score for the target sequence *Ta* was then calculated as follows:

(7) $$B\;Epic\;Score_{Ta}=median\left(s_{Ta}\right)$$

where *B* *Epic* *Score_Ta_* is the median B‐Epic Score of the set *s_Ta_*, which consists of 15‐mer peptides derived from the target peptide *Ta*.

To mitigate potential anomalies and visually display B‐cell activation distribution of long sequences intuitively, adjacent peptides were used to adjust the B‐Epic Score of the target peptide. Notably, the Sliding B‐Epic Score requires a contextual background of the target peptide (i.e., flanking adjacent sequences) for its calculation.

For each target peptide positioned at position *i* within the long sequence, the average was computed B‐Epic Score encompassing the target peptide and its two adjacent 15‐mer peptides in both N‐ and C‐terminal directions (Figure S1G, Supporting Information). This method, established by Santiago J. Carmona,^[^ ^16^ ^]^ provided a more robust indicator for assessing B‐cell activation by accounting for local sequence context. Specifically, for a target peptide located at position *i* of the long sequence, its Sliding B‐Epic Score was calculated as follows:

(8) $$Sliding\;B\;Epic\;Score_{Ta}=\frac{\sum_{i-2}^{i+2}B\;Epic\;Score_{i}}{5}$$

where *B* *Epic* *Score_i_* denotes the B‐Epic Score for the peptide at position *i*. The *Sliding* *B* *Epic* *Score_Ta_* of target peptide is computed as the average B‐Epic Score of 15‐mer peptides (*i* − 2 to *i* + 2).

The AUCs (area under the receiver operating characteristic curve) of Sliding B‐Epic Score with window sizes of 3, 5, and 7 AAs exhibited slight differences in the peptidome derived from 457 proteins of *T. cruzi* (Table S1, Supporting Information). When plotting Sliding B‐Epic Score distribution profiles, the choice of window size directly impacted the balance between smoothing effect and trend preservation. Due to an insufficient denoising process, using a smaller window (e.g., 3) resulted in a less smooth curve; in addition, the curve became overly smooth when using a larger window (e.g., 7), suppressing trend significance. Hence, a window size of 5 for the Sliding B‐Epic Score was a reasonable choice that balanced both considerations.

### Protein Structure Analysis

The 3D protein structures of VACA1 (AF‐Q48247‐F1‐model_v4.pdb) were downloaded from the AlphaFold Protein Structure Database (alphafold.ebi.ac.uk).

### Software

Data analysis was performed in R v4.1.1 using specialized packages for biological sequence analysis (Biostrings), data manipulation (dplyr, tidyr, data.table), and visualization (ggplot2, ggsci, ggrepel, patchwork). Machine learning and deep learning implementations utilized Python 3.9 with Transformer, scikit‐learn, PyTorch, and Biopython libraries. Prism v10.1 and R v4.1.1 (along with its corresponding packages, including imputeLCMD, pROC, and fields) were used for statistical analyses.

### Software–BepiPred‐1.0

For BCE prediction, BepiPred‐1.0 from the IEDB's integrated “bcell_standalone” library was employed. The algorithm generated per‐amino acid scores indicating BCE probability, with epitope likelihood determined by the median score across the target peptide (using default parameters).^[^ ^64^ ^]^

### Software–BepiPred‐3.0

Analysis was conducted in a CUDA 11.3‐enabled Docker environment. The algorithm utilized ESM‐2 matrices to generate Linear Epitope Score. The high‐precision “vt_pred” mode was implemented with a variable threshold while keeping other default parameters unchanged. Linear BCE probability was assessed using median Linear Epitope Scores.^[^ ^22^ ^]^

### Software–TMHMM

Transmembrane topology predictions were conducted using TMHMM v2.0c, which employs hidden Markov models to identify transmembrane helices. The output designates “o” for extracellular, “M” for transmembrane, and “i” for intracellular regions.^[^ ^65^ ^]^

### Software–PyMOL

Structural visualization was generated using PyMOL, with peptides highlighted according to their B‐Epic Score: red (B‐Epic Score > 0.35 and Sliding B‐Epic Score > 0.25), yellow (B‐Epic Score > 0.25 and Sliding B‐Epic Score > 0.15), and blue (remaining residues).

### Software–DIAMOND

Sequence similarity searches were conducted using DIAMOND against UniProt‐reviewed prokaryotic and *H. sapiens* proteomes.^[^ ^66^ ^]^ Filtering criteria were as follows: e‐value ≤ 1e^−3^ and 30% coverage for protein sequences; and e‐value ≤ 1 and 20% coverage for 15‐mer peptides. Foreignness Score was computed as the negative Bit‐Score from DIAMOND, with missing values imputed using a Gaussian distribution (µ = 250, σ = 100).

### Software–CD‐HIT

Sequence redundancy was eliminated using CD‐HIT with a 0.5 sequence similarity threshold.^[^ ^67^ ^]^

### Software–NetMHCIIpan

MHC class II binding predictions were performed using NetMHCIIpan against classical HLA‐DR, HLA‐DQ, and HLA‐DP alleles. Binding affinity was quantified using the EL Score, with values > 0.25 considered significant. The Max EL Score was defined as the max EL Score across the three classical alleles.^[^ ^68^ ^]^

### False Discovery Rate (FDR)

The calculation of FDR began by constructing a kernel density estimation (KDE) function using the negative data from the training/testing dataset. Then, the cumulative probability of the target value was computed using this KDE function, and the *P* value was obtained by subtracting the cumulative probability from 1, indicating whether the target value lay in the right tail of the KDE distribution. The FDR was controlled using the Benjamini‐Hochberg (BH) procedure, which sorted the *P* values and compared each *P* value with a threshold calculated based on the rank of the *P* value. This procedure determined which hypotheses were considered significant while controlling the false discovery rate. The critical value is typically set to 0.05, with the goal of ensuring that the false positive rate is less than 5%.

(9) $$Threshold=\frac{i}{m}*Q$$

*i* is the rank of the *i^th^P* value after sorting (from smallest to largest). *m* is the total number of tests (i.e., the number of *P* values). *Q* is the given FDR threshold (typically set to 0.05).

### Peptide Synthesis

From the B‐Epic predicted repertoire, 9 vaccine candidate peptides (VCPs) and 1 negative control (NC) were selected Table S2, Supporting Information). Each 15‐mer peptide (purity > 95%) was synthesized with terminal cysteine residues to enable directional conjugation. The peptides were conjugated to either keyhole limpet hemocyanin (KLH) or bovine serum albumin (BSA) via maleimide‐thiol chemistry, with carriers chosen based on experimental requirements. KLH conjugates (*n* = 10) were used for immunization protocols, while BSA conjugates (*n* = 10) were used for ELISA. For human serum analysis, four BSA‐conjugated EBNA1 peptides were synthesized (Table S2, Supporting Information). GenScript Biotech Corporation (Nanjing, China) performed all peptide synthesis, carrier protein conjugation, and quality control analyses under good laboratory practice (GLP) conditions.

### Patient Inclusion and Exclusion Criteria

Eligible participants were adults aged 18 or older with histopathologically confirmed primary nasopharyngeal carcinoma (NPC) who had not received prior antitumor therapies or palliative treatment. The study included participants regardless of gender or tumor stage. Cases with unclear pathological diagnoses, incomplete clinical documentation, non‐keratinizing or basaloid squamous cell carcinoma subtypes, benign nasopharyngeal lesions detected by endoscopy, or secondary malignancies confirmed by imaging (computed tomography [CT], magnetic resonance imaging [MRI], or positron emission tomography‐CT [PET‐CT]) were excluded.

Healthy controls (HCs) were recruited from the Health Examination Center of Sun Yat‐sen University Cancer Center (SYSUCC). These individuals showed no signs of primary NPC during physical examinations, hematological testing, tumor biomarker analysis, or imaging evaluations. They had no history of nasopharyngeal disorders at enrollment and remained free of NPC throughout a minimum 12‐month follow‐up period. The study was approved by the Ethics Committee of Sun Yat‐sen University Cancer Center (Ethics Approval No.: B2024‐673‐01).

### Animal Models

C57BL/6 mice (8–10 weeks old, male) were maintained under specific pathogen‐free (SPF) conditions with controlled temperature (22 ± 1 °C), humidity (55 ± 10%), and 12 h light/dark cycles. All animal experiments were conducted in accordance with protocols approved by the Institutional Animal Care and Use Committee of Sun Yat‐sen University Cancer Center (Ethics Approval No.: L025504202504016).

### Immunization

Mice received primary subcutaneous immunization in the inguinal region with KLH‐conjugated peptides emulsified in CpG (cytosine phosphorothioate guanine) oligodeoxynucleotide adjuvant (100 µg per mouse, 1:1 w/v) or vehicle control (PBS). Booster immunizations were administered at 7 day intervals for 3 consecutive weeks. Blood samples were collected via tail vein puncture at day 31 post‐primary immunization.

### Tissue Collection and Processing

At the experimental endpoint (day 45), mice were humanely euthanized via gradual exposure to 4% isoflurane. Inguinal lymph nodes were harvested and processed either for flow cytometry analysis or histological examination. For flow cytometry, tissues were enzymatically dissociated using Collagenase IV (1 mg mL^−1^; Gibco, Cat. No.: 17 104 019) and DNase I (0.1 mg mL^−1^; Gibco, Cat. No.: 18 047 019) in RPMI‐1640 medium at 37 °C for 25 min. Single‐cell suspensions were obtained following red blood cell lysis. For histological analysis, tissues were fixed in 4% paraformaldehyde overnight, dehydrated through graded ethanol, embedded in paraffin, sectioned, and processed for immunostaining.

### Enzyme‐Linked Immunosorbent Assay

Antigen‐specific antibody responses were evaluated using indirect ELISA. Briefly, 96‐well plates were coated with BSA‐conjugated peptide (5 µg mL^−1^ in coating buffer; Solarbio, Cat. No.: C1055) overnight at 4 °C. After blocking with 2% BSA in PBS, serial dilutions of mouse sera (1:20 to 1:320) were added and incubated for 1 h at 37 °C. All samples at each dilution level were analyzed in triplicate. HRP‐conjugated goat anti‐mouse IgG (1:5000; Abcam, Cat. No.: AB205719) was used as the secondary antibody. Signal was developed using TMB substrate (Beyotime, Cat. No.: P0206) and quantified at 450 nm using a BioTek Epoch microplate reader.

### Human Serum Analysis

Clinical serum samples were diluted at a 1:30 ratio in PBS and analyzed using microplates pre‐coated with synthetic soluble EBNA1 15‐mer peptides. Antibody binding was detected using HRP‐conjugated goat anti‐human IgG (1:10000; Abcam, Cat. No.: AB6858). Optical density was measured at 450 nm using a BioTek Epoch microplate reader.

### Flow Cytometry Analysis

Single cell suspensions were prepared from lymph nodes in ice‐cold FACS buffer (PBS supplemented with 1% BSA and 0.1% sodium azide). To block non‐specific binding, cells were incubated with Rat anti‐mouse CD16/32 Fc receptor blocking antibody (1 µg mL^−1^; BD Biosciences, Cat. No.: 553 142) for 10 min at 4 °C. For surface marker analysis, cells were stained with fluorochrome‐conjugated antibodies (Biolegend) as follows: Alexa Fluor 700 Rat anti‐mouse CD3ε (17A2), FITC Rat anti‐mouse CD4 (GK1.5), PerCP/Cy5.5 Rat anti‐mouse CD19 (6D5), PE/Cyanine7 Mouse anti‐mouse CD95 (SA367H8), PE/Dazzle 594 Rat anti‐mouse PD‐1 (29F.1A12), BV605 Rat anti‐mouse CXCR5 (L138D7), APC/Fire 750 Rat anti‐mouse ICOS (C398.4A), BV510 Rat anti‐mouse/human CD45R/B220 (RA3‐6B2), and APC Rat anti‐mouse/human GL7 (GL7). Antibodies were diluted 1:500 in PBS containing 1% BSA (w/v). Surface staining was performed for 15 min at 4 °C in the dark. The flow cytometry experiments were conducted at the Core Facility of the State Key Laboratory of Oncology, Sun Yat‐sen University Cancer Center (SYSUCC). Flow cytometric analysis was conducted using a CytoFLEX LX (Beckman Coulter), and data were analyzed using FlowJo v10 software (BD Biosciences). Germinal center (GC) B cells were defined as CD3^−^CD4^−^CD19^+^CD95^+^B220^+^GL7^+^ populations, while T follicular helper (Tfh) cells were identified as CD4^+^PD^−^1^+^ICOS^+^CXCR5^+^ populations.

### Immunofluorescence Microscopy

Lymph nodes were fixed in 4% paraformaldehyde for 16 h at room temperature, followed by dehydration through a graded ethanol series, paraffin embedding, and sectioning (4 µm thickness). Multiplexed immunofluorescence staining was performed using the PANOVUE kit (Cat. No.: TSA‐RM‐827258B) according to the manufacturer's instructions. Following antigen retrieval, sections were incubated with primary antibodies overnight at 4 °C: Rabbit anti‐mouse CD8a (ABclonal, Cat. No.: A23305PM), Rabbit anti‐mouse CD19 (Cell Signaling Technology, Cat. No.: 90 176), and Biotin Rat anti‐mouse PNAd (BioLegend, Cat. No.: 120 804). Secondary detection was achieved using HRP‐conjugated Goat anti‐rabbit IgG (PANOVUE, Cat. No.: 10 506 001 060). Nuclei were counterstained with DAPI (SouthernBiotech, Cat. No.: 0100–20).

## Conflict of Interest

The authors declare no conflict of interest.

## Author Contributions

J.Z.L., Y.T.W., C.S. contributed equally to this work. J.P.L. and M.S.Z. conceived the study and revised the manuscript; J.Z.L. constructed the B‐Epic model; Y.T.W. and C.S. conduct the key experiments; J.Z.L., Y.T.W. and C.S. processed the data, drew the figures, drafted and edited the manuscript; J.Z.L., L.P.C. facilitated the bioinformatic analysis; T.L. performed the immunohistochemical staining; Z.F.W. performed FCM experiment and analysis; L.N.C and P.L.L. performed ELISA experiments and analysis of human samples. Q.Z., B.G., Y.C., Z.K.L., C.G.Z., B.Y.L., Q.Z., provided vital suggestions for this work. All authors have read and approved the final version of the article.

## Supporting information



Supporting Information

Supporting Information

Supplemental Table1‐12

## Data Availability

The data that support the findings of this study are available from the corresponding author upon reasonable request.
