# Supplementary material for: B‐EPIC: A Transformer‐Based Language Model for Decoding B Cell Immunodominance Patterns
Source: Adv Sci (Weinh). 2025 Oct 7;12(47):e08896. doi: 10.1002/advs.202508896 (PMC12713022; doi:10.1002/advs.202508896)
Supplement: Supplementary file 1 — Supporting Information [file ADVS-12-e08896-s003.pdf]

Figure S1

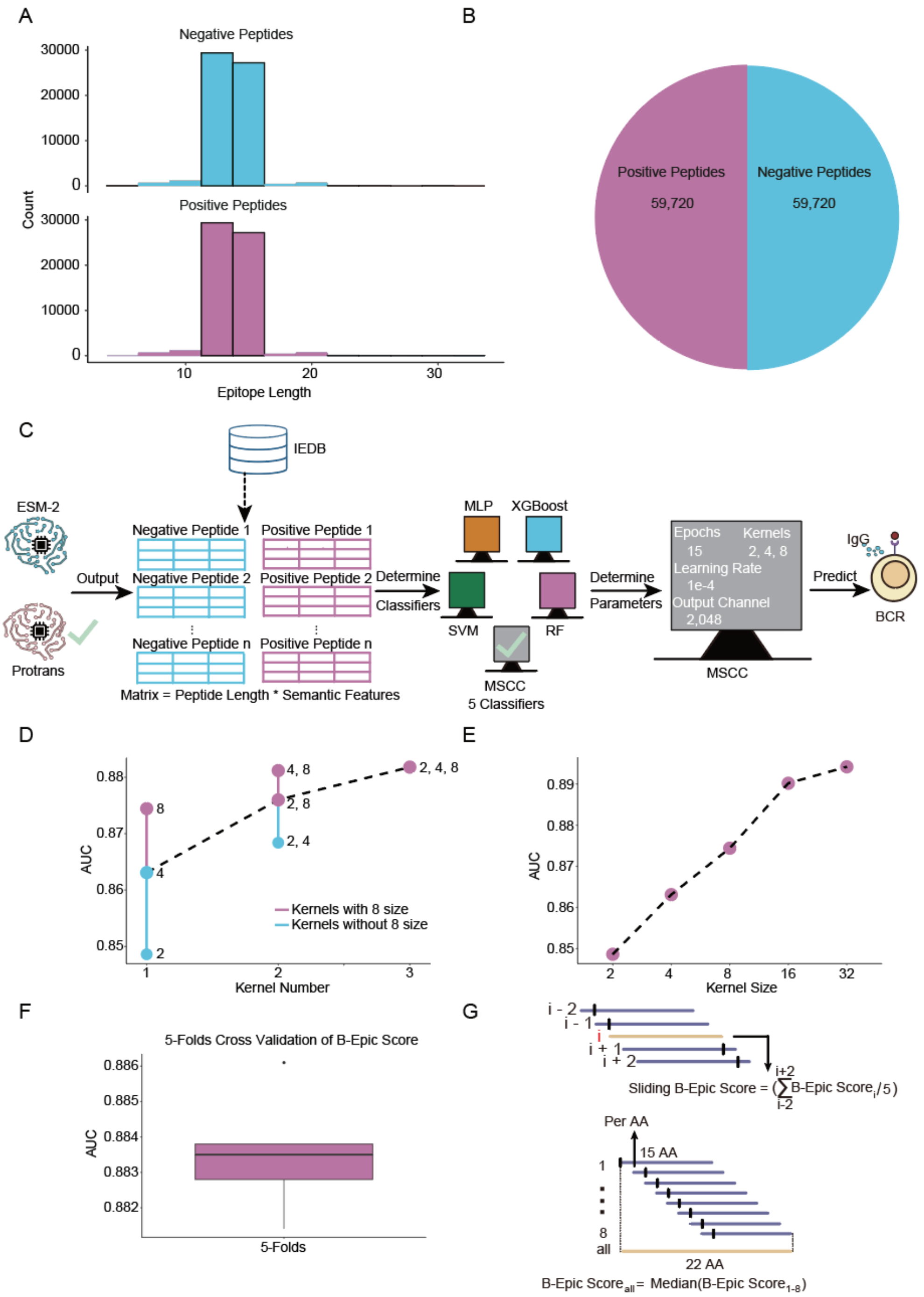

Figure S1 Development of B-Epic: a Transformer-based Approach for BCE Prediction. A) The length distribution of peptides in IEDB with corresponding positive or negative B cell activation experimental results was shown. IEDB contained the largest number of peptides with lengths ranging from 14 to 16 AAs. B) The ratio of

peptides with positive (n = 59,720) and negative (n = 59,720) B cell activation experimental results was 1:1. C) The construction process of B-Epic was illustrated, specifically ranging from semantic embedding generation to classifier determination. D) MSCCs with different kernel sizes and numbers were presented. Pink and blue represented kernels with versus without size 8, respectively. Each data point corresponded to a specific kernel configuration. E) The AUCs for MSCC with different kernel sizes (ranging from 1 to 32, corresponding to the maximum input length) were shown. F) The schematics of calculation methods for the Sliding B-Epic Score (top) and the B-Epic Score for long sequences (exceeding 15 AAs; bottom) were displayed. G) The AUCs of B-Epic in 5-folds cross-validation were presented.

Figure S2

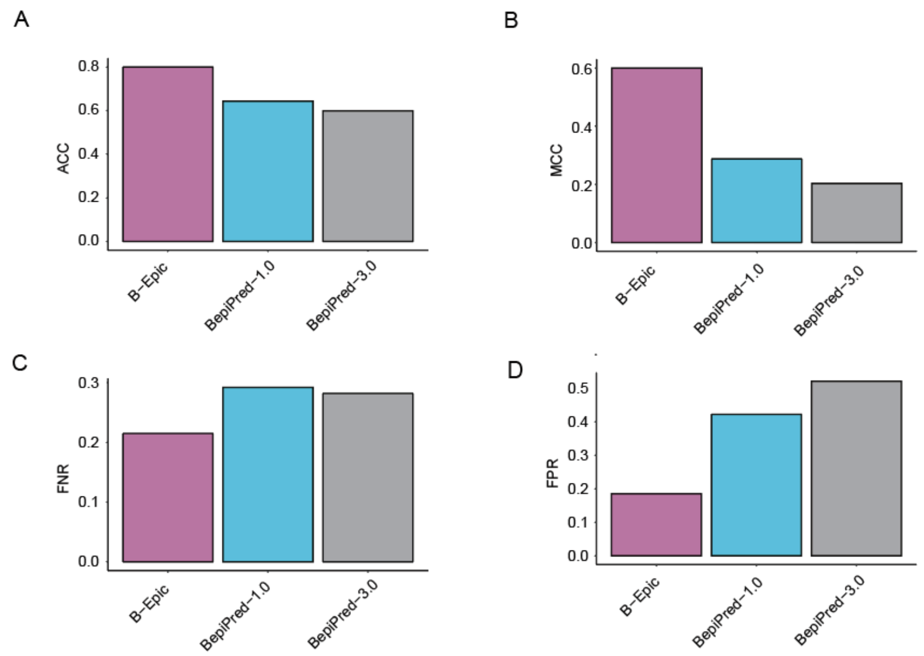

Figure S2 B-Epic exhibited high accuracy evaluated through multiple indexes in IEDB testing dataset.

A-D) The ACC (A), Matthews' coefficient (MCC; B), FNR (C), and FPR (D) of B-Epic, BepiPred-1.0, and BepiPred-3.0 in the IEDB testing dataset were shown

Figure S3

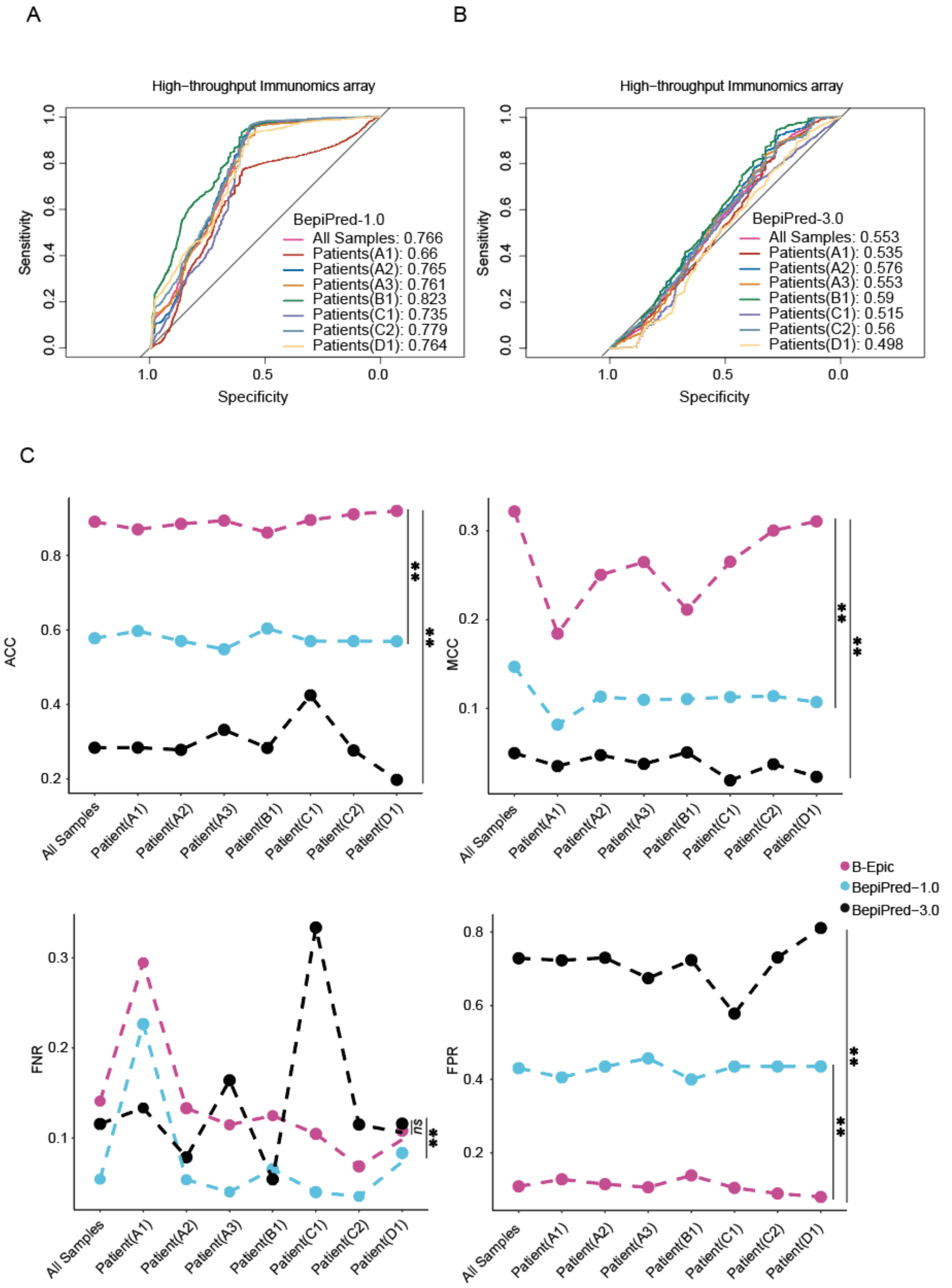

Figure S3 B-Epic Identifies Immunoreactive Peptides from Peptidome of *T. cruzi*.

A, B) The AUCs of BepiPred-1.0 and BepiPred-3.0 for screening immunoreactive

peptides in the peptidome of *T. cruzi* were shown. C) The ACC, MCC, FNR, and FPR of B-Epic, BepiPred-1.0, and BepiPred-3.0 in immunomic arrays of *T. cruzi* were presented. Statistical significance was denoted as follows: **P* < 0.05; ***P* < 0.01; ****P* < 0.001; *****P* < 0.0001; ns (not significant). The significance level (α) was set at 0.05. Statistical analyses were performed using the two-tailed Mann-Whitney U test (Figure S3C).

Figure S4

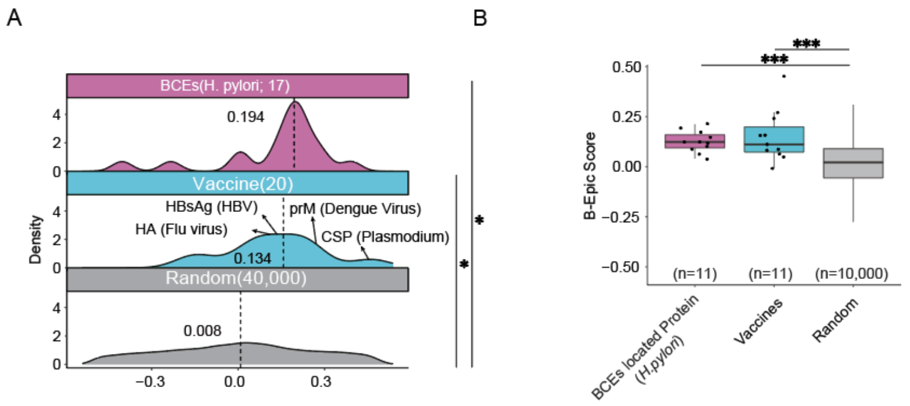

Figure S4 BCEs of *H. pylori* with experimental evidence had higher B-Epic Score. A) The density of B-Epic Score for BCEs in *H. pylori* (n = 20), vaccine targets (proteins and peptides; n = 20), and random sequences (n = 40,000) was shown. B) The box plot displayed the B-Epic Score (median ± IQR) of BCE-located proteins in *H. pylori* (n = 11), vaccine targets (proteins; n = 11), and random proteins (n = 10,000). Statistical significance was denoted as follows: **P* < 0.05; ***P* < 0.01; ****P* < 0.001; *****P* < 0.0001; ns (not significant). The significance level (α) was set at 0.05. Statistical analyses were performed using the two-tailed Mann-Whitney U test (Figure S4 A, B).

Figure S5

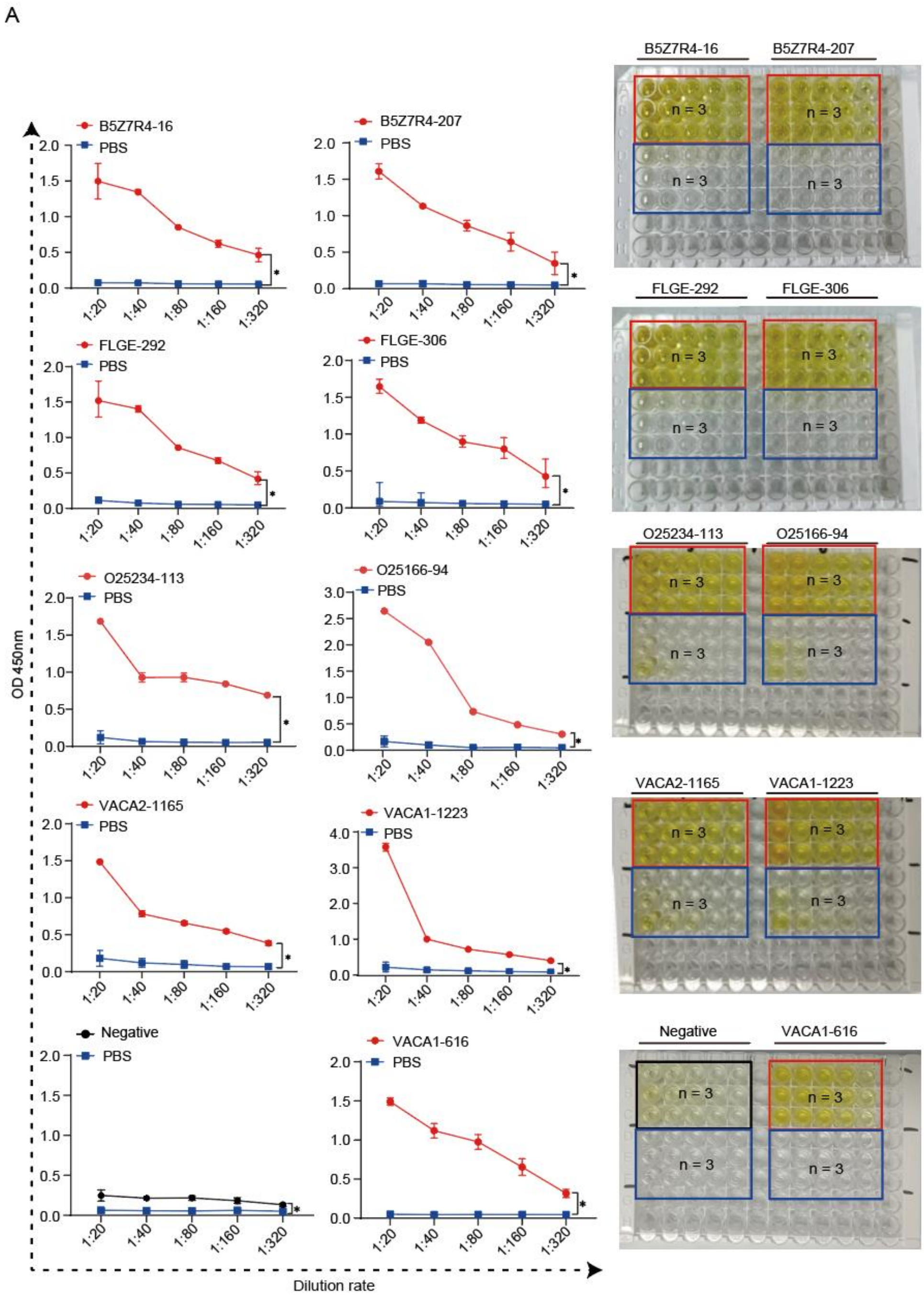

Figure S5 ELISA detected antibody level in sera of VCP-immunized mice. A) Corresponding mouse sera (3 replicates) were diluted into 5 different concentrations (1:20 to 1:320) and sequentially incubated with the synthetic peptides. Binding results were then detected by measuring the OD_{450nm} after color development. Data were

presented as the mean ± SD (A; some small SDs not visually distinguishable). Statistical significance was denoted as follows: *P < 0.05; **P < 0.01; ***P < 0.001; ****P < 0.0001; ns (not significant). The significance level (α) was set at 0.05. Statistical analyses were performed using the two-tailed t test (Figure S5A).

Figure S6

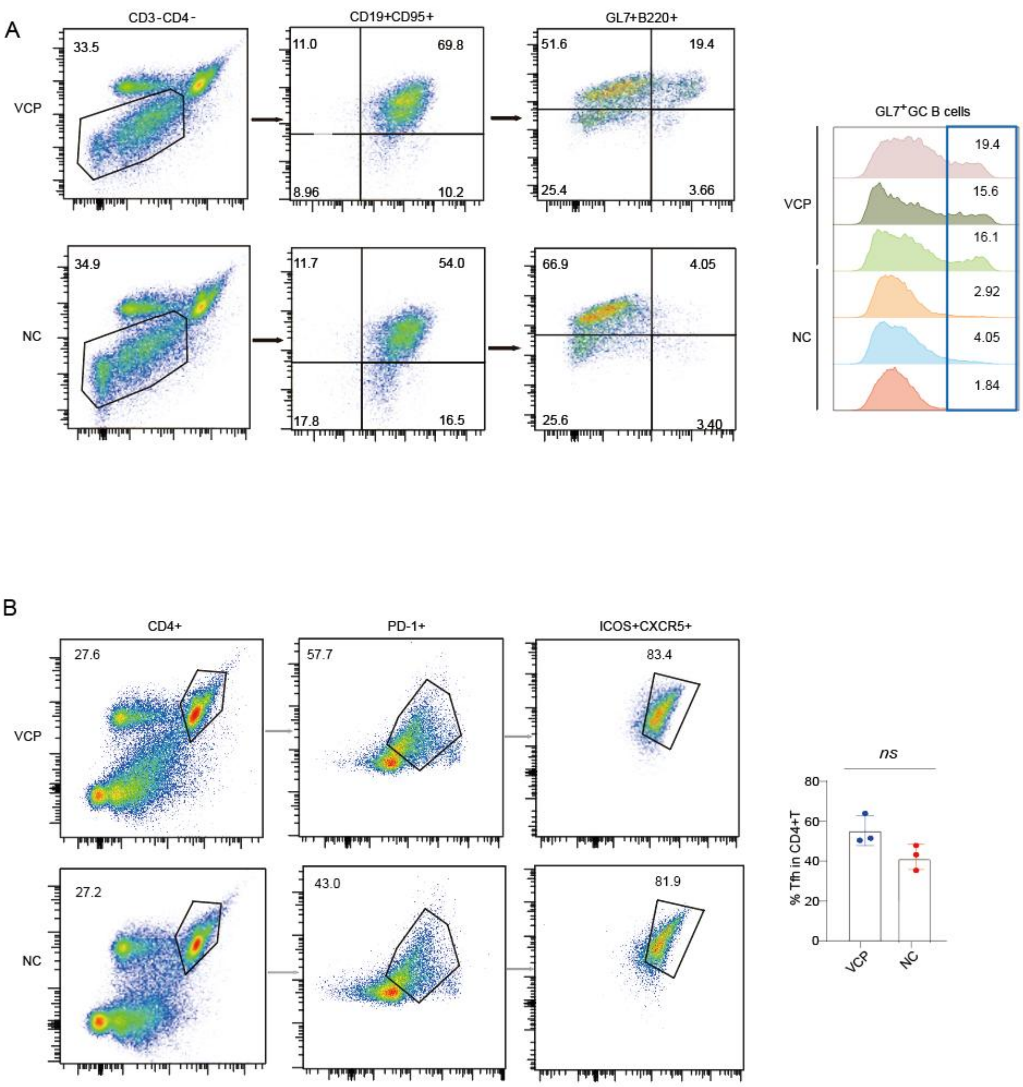

Figure S6. The gating strategy used to identify GL7$^+$ and B220$^+$ germinal center (GC) B cells in mice lymph node. A) CD3$^-$ and CD4$^-$ were used to identify B cells, followed by gating with CD19 and CD95. Subsequently, GL7$^+$ and B220$^+$ cells were

confirmed as GC B cells. Both VCPs (up) and NC (down) used same gating strategy. B) Follicular helper T (Tfh) cells were defined as $CD4^+$ T cells expressing PD-1, with subsequent sequential gating for ICOS and CXCR5. The proportion of Tfh cells within $CD4^+$ T cells was quantified and compared between mice immunized with the VCPs and those with the NC. Data were presented as the mean ± SD (B). Statistical significance was denoted as follows: *$P < 0.05$; **$P < 0.01$; ***$P < 0.001$; ****$P < 0.0001$; ns (not significant). The significance level (α) was set at 0.05. Statistical analyses were performed using the two-tailed t test (Figure S6B).

Figure S7

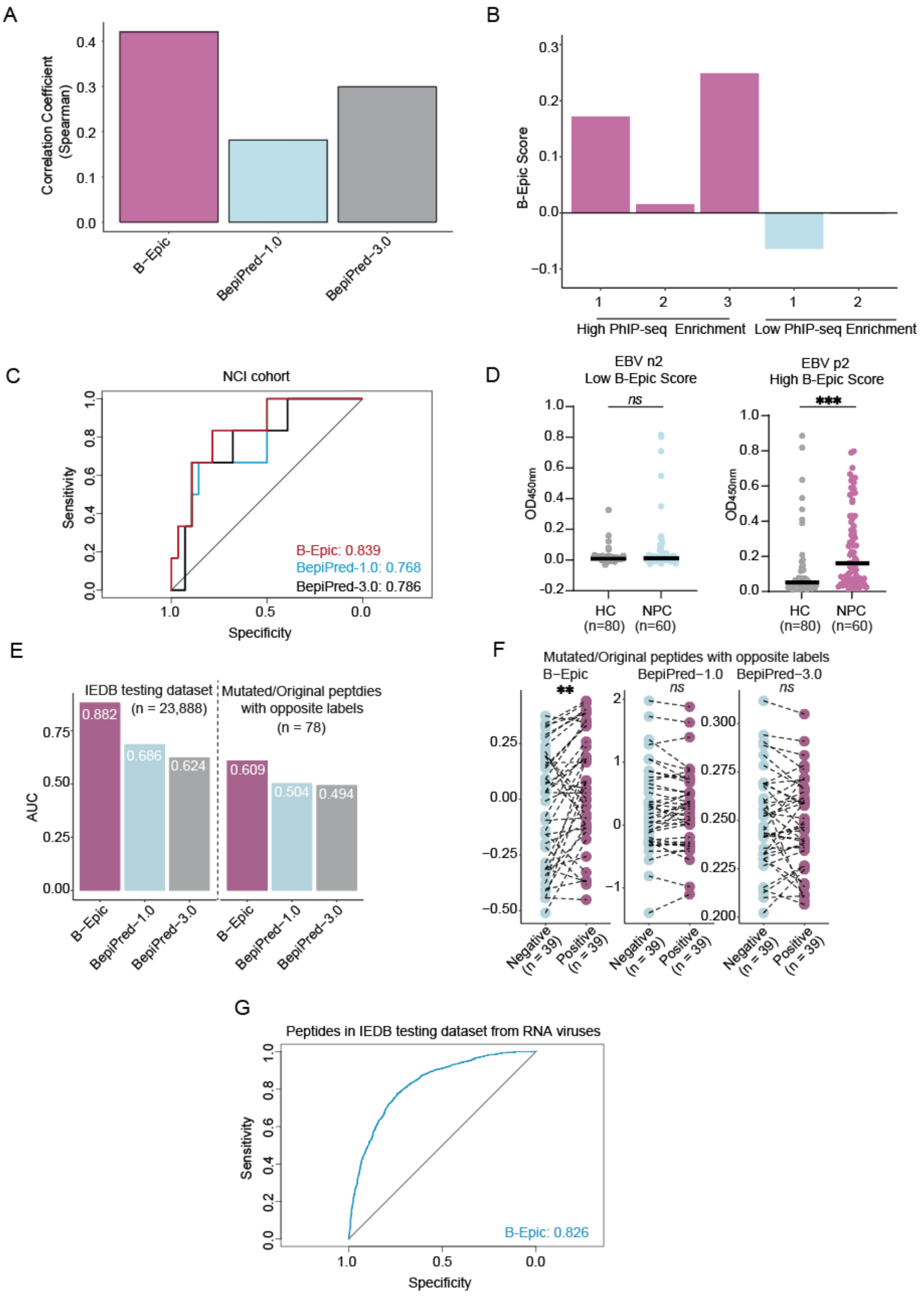

Figure S7. B-Epic Exhibited Exceptional Performance on Identifying Pan-Immunoreactive Peptides of EBV in Large Clinical Cohort. A) The Spearman correlation between predictive results of the three tools and median enrichment (calculated across 899 samples) of sequences in the PhIP-seq cohort were shown. B)

The B-Epic Score of three long peptides with high PhIP-seq enrichment and two with low enrichment were presented. In addition, the B-Epic Score of Peptide 2 with low PhIP-seq enrichment was nearly 0 and thus cannot be visualized. C) B-Epic presented a higher AUC than BepiPred-1.0 and BepiPred-3.0 for screening PhIP-confirmed BCEs. D) Peptides with high B-Epic Score exhibited higher levels of corresponding specific IgG expression in NPC patients (n = 60) than in HCs (n = 80). The black line represented the median expression in NPC patients or HCs. E) A bar plot presented the AUCs of B-Epic, BepiPred-1.0, and BepiPred-3.0 in the IEDB testing dataset and for mutated/unmutated peptides (n = 2,042) with opposite B cell activation experimental results. F) A faceted paired scatter plot presented the predictive results of the mutated/unmutated peptides with opposite B cell activation experimental results in IEDB, as calculated by B-Epic, BepiPred-1.0, and BepiPred-3.0. The x-axis represented the B cell activation experimental results of these peptides. G) The AUC of B-Epic in the peptidome from the IEDB testing dataset of RNA viruses (n = 3,287) was shown. Statistical significance was denoted as follows: *$P < 0.05$; **$P < 0.01$; ***$P < 0.001$; ****$P < 0.0001$; ns (not significant). The significance level (α) was set at 0.05. Statistical analyses were performed using the pair two-tailed Mann-Whitney U test (Figure S7F).
